# Associations between violence in childhood, depression and suicide attempts in adolescence: evidence from a cohort study in Luwero district, Uganda

**DOI:** 10.1186/s12889-024-20950-7

**Published:** 2024-12-18

**Authors:** Rebecca Akunzirwe, Daniel J. Carter, Lauren Hanna, Anja Zinke-Allmang, Aggrey Akim, Simone Datzberger, Jenny Parkes, Louise Knight, Lydia Atuhaire, Janet Nakuti, Angel Mirembe, Elizabeth Allen, Dipak Naker, Karen Devries, Amiya Bhatia

**Affiliations:** 1 Raising Voices, Kampala, Uganda; 2 Department of Epidemiology and Population Health, London School of Hygiene and Tropical Medicine, London, UK; 3 Department of Social Policy and Intervention, University of Oxford, Oxford, UK; 4 Institute of Education, University College London, London, UK

**Keywords:** Violence against children, Child health, Adolescent health, Suicide, Mental health, LMICs, Longitudinal, Cohort

## Abstract

**Background:**

Many studies have documented an association between violence victimisation and poor mental health. However, few studies use longitudinal data from low- and middle-income countries with attention to how associations differ by experiencing specific types of violence or alongside different contexts of peer and family support. In this study, we quantify the association between experiences of violence in early adolescence and depression and suicide attempts in late adolescence and explore whether this association is modified by family and peer connectedness.

**Methods:**

Data came from the Contexts of Violence Against Children (CoVAC) cohort study in Luwero District, Uganda, involving 2773 participants aged 11–14 years at Wave 1 (2014) and 15–18 years at Wave 2 (2018). Physical, sexual, and emotional violence were measured at Wave 1. Mental health outcomes, depression symptoms in the past two weeks, and lifetime suicide attempts were measured at Wave (2) We used logistic regression models, stratified by sex, to estimate adjusted odds ratios with an interaction term to test for effect modification by peer and family connectedness at Wave 1.

**Findings:**

At Wave 1, the prevalence of any violence from any perpetrator was 90% (physical violence: 87%, physical violence excluding caning: 68%, sexual violence: 6.3%, emotional violence: 56.8%). At Wave 2, 13.3% of participants had scores indicative of depression and 4.3% reported ever attempting suicide. Physical violence excluding caning, emotional violence, and sexual violence during early adolescence increased the odds of depression and attempting suicide in late adolescence for both boys and girls. Experiencing any violence (including caning) in early adolescence was not associated with depression in late adolescence, including in sex-stratified models. Childhood experience of any violence was associated with a suicide attempt violence in early adolescence (aOR: 2.60; 95%CI: 1.08, 6.27). High peer support mitigated the effect of any violence and physical violence on depression.

**Conclusions:**

Findings highlight the importance of efforts to prevent violence and improve access to response and support services for violence and mental health for young people. Findings also underscore the important role friends and peer networks can play in mitigating the effects of violence as young people grow up.

**Supplementary Information:**

The online version contains supplementary material available at 10.1186/s12889-024-20950-7.

## Introduction

One in seven children and adolescents (14%) globally live with a mental health disorder and in 2019 the Global Burden of Disease study found poor mental health to be the 7th leading cause of years lived with disability globally [1]. The highest burden of poor mental health occurs in low and middle-income countries (LMIC), including those in Africa, where an estimated 37 million children and adolescents are affected by suicide ideation, anxiety, bipolar disorder, and depression. Uganda has some of the highest prevalence of mental health disorders in children and adolescents in Africa: a UNICEF analysis of Institute for Health Metrics and Evaluation (IHME) data reported a 12.5% prevalence of mental health disorders (depression, anxiety, bipolar, eating disorder, autism spectrum, conduct disorder, schizophrenia, idiopathic intellectual disability, attention-deficit/hyperactivity disorder and a group of personality disorders) among young people aged 10–19 years [2]. Mental health disorders experienced in childhood can extend into adulthood with more severe presentation, especially if untreated [3]. Poor mental health can also increase the risk of suicide [4]. UNICEF estimates suggest twenty adolescents die from suicide every day globally with boys accounting for about twice the number of deaths compared to girls [2]. Country estimates on the prevalence of suicide among adolescents in Uganda are limited.

Studies in high- and low-income countries, including Uganda, show that the factors that affect depression and suicide in adolescence and young adulthood exist across the levels of the social ecology and include political instability, unemployment, low family cohesion, intergenerational, genetic and epigenetic factors [5, 6], internalising problems in childhood, and experiences of violence, among others [7–12]. Many cross-sectional and cohort studies in high-income countries (HIC) have specifically explored links between experiencing violence and mental health and find that violence is associated with anxiety, depression, suicidal ideation, and suicide attempts in adulthood [13–19].

Violence against children (VAC) can include physical, emotional, or sexual violence, neglect or maltreatment, bullying, sexual exploitation, and online violence among others [20]. VAC may be perpetrated by caregivers, school staff, peers, intimate partners, and/or strangers [21]. Despite its importance as both a human rights and public health issue of concern, VAC continues to be highly prevalent worldwide. In 2016, more than half of children globally experienced at least one form of violence in the past year [22]. According to the 2021 estimates from the African Partnership to End Violence against Children, across all of Africa, more than 50% of children had experienced physical violence [23]. Data from the 2018 Violence Against Children Survey in Uganda provided some of the first nationally representative estimates of prevalence and showed that 35% of young women and 17% of young men experienced sexual violence in their childhood; about 33% of young women and men experienced emotional violence in their childhood and about 59% of young women and 68% of young men reported experienced harsh forms of physical violence in their childhood, such as kicking, punching, whipping, strangling, smothering, trying to drown, smothering, beating with an object excluding caning [24].

Although there is strong evidence linking experiences of violence to poor mental health, most studies use cross-sectional data which precludes an examination of the timing of these associations. In addition, few studies examine factors within the social ecology that can modify links between violence, mental health, and suicidality. Existing studies have come primarily from HICs and report that peer and family support buffer the negative consequences of experiences of violence on mental health [25–27]. Although evidence from LMICs is limited, a few studies also show the moderating role of peer and familial connectedness and support on the association between experience of violence and externalising or internalising problems [28, 29].

In this study, we draw on longitudinal data to examine the impact of childhood exposure to different forms of violence on depression and suicide attempts in later adolescence and young adulthood. Therefore, this study has three aims: first, to determine the burden of experiencing depression and suicide in adolescence; second, to quantify the association between early adolescent experiences of violence and late adolescent depression symptoms and suicide attempts; and third, to explore whether and by how much family and peer connectedness and support in childhood modifies this hypothesised association between early adolescence violence and late adolescence mental health outcomes.

## Methods

### Study design and setting

Data come from the Contexts of Violence Against Children (CoVAC) cohort study. The study design is described in detail elsewhere [30]. Briefly, CoVAC is a prospective longitudinal mixed-methods study in Luwero district, Uganda, to explore how individual and contextual characteristics encourage or interrupt the association between experiences of violence in early adolescence and social and health outcomes in later adolescence. The original sample included students in primary school, grades 5 to 7, recruited from 42 schools in Luwero District. Quantitative data were collected at three time points: adolescents (11–14 years) recruited at Wave 1 in 2014, were followed up in 2018 at Wave 2 (15–18 years), and in 2021/2022 at Wave 3 (18–21 years). This analysis draws on data from Waves 1 (early adolescence) and 2 (late adolescence).

### Study participants and procedures

All adolescents who participated in the Good Schools Study (GSS) in 2014 and in Wave 1 and who agreed to be re-contacted were eligible for Wave 2 [30]. Of the 3431 participants (1578 males and 1853 females) recruited at Wave 1, 2 caregivers opted out, 14 adolescents refused to participate, 2 had cognitive difficulties, 19 died, 24 moved out of Uganda, 145 had moved to districts too far from the study site to follow-up at Wave 2, 447 were not contactable at Wave 2, and 3 had incomplete interviews at Wave 1. Thus, there were 2773 participants (80.8% of participants at Wave 1) in Wave 2 including 1328 males and 1445 females.

Raising Voices -- a non-profit feminist organization in Uganda working towards the prevention of violence against women and children -- [31], MRC/UVRI and LSHTM led all quantitative study procedures. The interview tool used to collect data was developed from internationally recognized tools [32]. All researchers received three to six weeks of training on how to conduct violence data collection with children and adolescents, build rapport, and ensure participant safety at Wave 1 and Wave 2. To re-contact participants at Wave 2, data collected at Wave 1 were used, including participant names, home addresses, and contact phone numbers.

To ensure data were collected with community support, a meeting with key stakeholders, including the Ministry of Education and headteachers, was held to share information with them regarding the study as well as to develop standardised information sheets and conversational scripts to explain the study (Wave 2). Headteachers of the original sample of schools in Wave 1 were contacted and informed about the study and that adolescents were being contacted. Adolescents not in school were traced using the available contact information, contacts at their last school, and in their communities. Researchers conducted in-person interviews with participants in a private place either near their school or at home. All participants were offered counselling and any participant who reported severe forms of violence was offered referrals to local services which were overseen and coordinated by the study counsellor and a local organisation. Following the interview, participants received soap worth 7,500 Ugandan shillings (~ USD 2 at the time of writing).

### Variables

#### Outcome: depressive symptoms and suicidality in late adolescence

We examined two mental health outcomes measured at Wave 2: symptoms of depression and suicide attempts. Depressive symptoms were measured according to the full Patient Health Questionnaire for Adolescents (PHQ-A) [33], a well-validated scale in resource-constrained settings with a Cronbach’s alpha of 0.84 [34]. The PHQ-A includes questions on whether, in the past 2 weeks, the adolescent felt down, depressed, irritable, hopeless, tired, or had little energy, amongst other questions. These responses were ranked on a Likert scale from 0 (not at all) to 3 (nearly every day) and participants could score anywhere from 0 to 27 across items. The total score was dichotomized in accordance with standard usage of PH9Q-A into no suspected depression (score: 0–9; reference group) and suspected depression (score: 10–27) [35–37].

Suicide attempts were measured by asking participants “Have you ever, in your whole life, tried to kill yourself or made a suicide attempt”. Suicide attempt responses were dichotomised into yes and no (reference group).

#### Exposure: lifetime violence in early adolescence

The main exposure variable was lifetime experience of the different types of violence at Wave 1. Violence perpetrated by caregivers, school staff, peers, intimate partners and any other child or adult was measured using questions adapted from the International Society for the Prevention of Child Abuse and Neglect Child Abuse Screening Tool-Child Institutional (ICAST-CI) [38]. Intimate partner violence (IPV) was measured by a tool adapted from the WHO Multi-Country study on women’s health and domestic violence against women [39], and the Conflict in Adolescent Dating Relationships Inventory (CADRI) questionnaire [40, 41]. Violence items were categorized into any physical violence (measured using 37 items), physical violence excluding caning due to the high prevalence of caning (measured using 36 items), emotional violence (measured using 20 items), and sexual violence (measured using 34 items) (Supplementary Table S1). We categorized experience of any form of physical, emotional, or sexual violence as ‘any violence’ and any experience of physical excluding caning, emotional or sexual violence as ‘any violence excluding caning’. Binary variables were created for each violence variable (0 = no or no response, 1 = yes to at least one item) (Table S1).

#### Effect modifiers: sex, family and peer connectedness

Family and peer connectedness were measured at Wave 1 and questions were adapted from the Minnesota Student Survey and Add Health tool [42]. Family connectedness was measured using five items (feeling that parents or caregivers care; feeling safe at home; feeling of belonging at home; like spending time at home and being scared of parents/caregivers). Peer connectedness was measured using three items (feeling close to students at school; having friends to talk to about important things; having friends they could count on for support). Participants responded to the item-level questions using a four-point scale (never, sometimes, most of the time, all of the time). A total score was obtained for each of the connectedness variables by summing the Likert responses to each individual item and a binary variable was created for high or low connectedness by dichotomising the total score based on the median score [43].

### Covariates

**Fig. 1:**
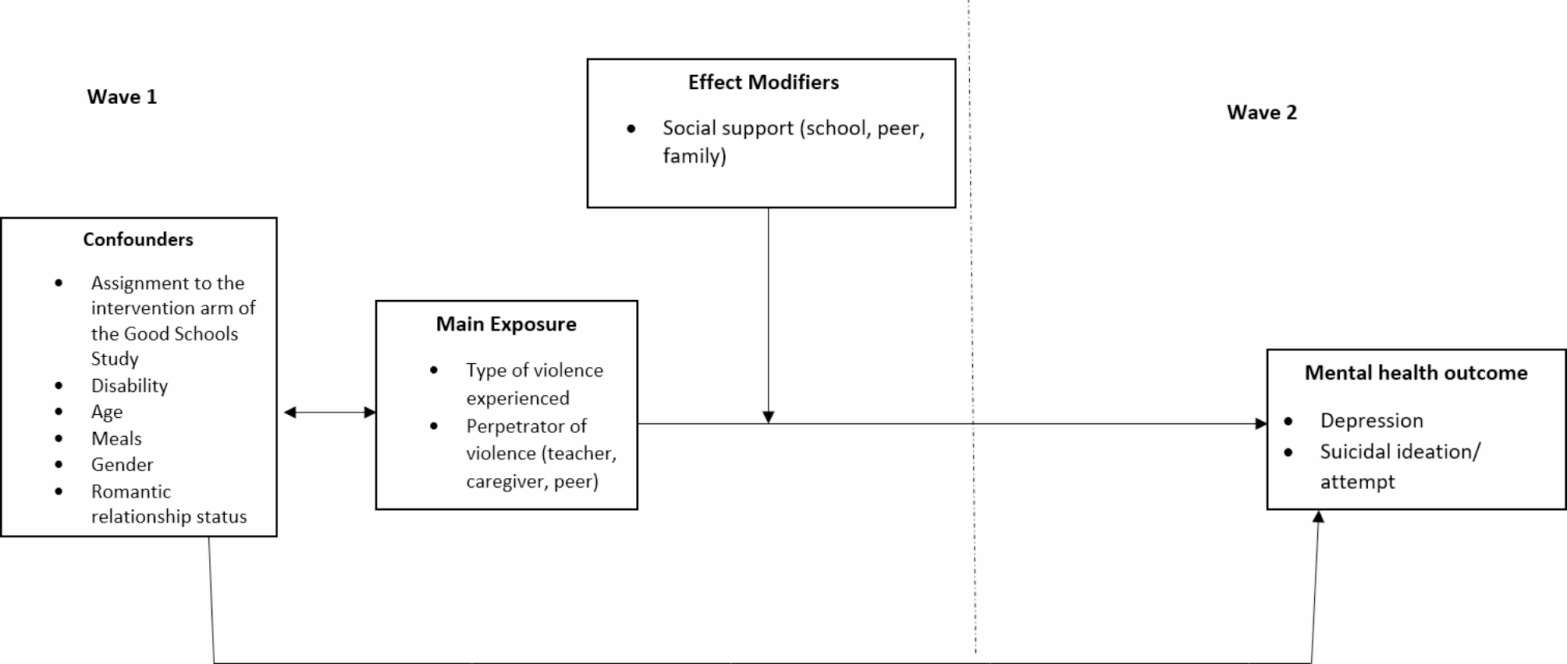
A directed acyclic graph showing the pathways and interactions between violence experienced at Wave 1 and mental health outcomes at Wave 2

We identified age, sex, assignment to the intervention arm of GSS at Wave 1, the daily number of meals, romantic relationship status, and disability as confounders, and family and peer connectedness as effect modifiers. These covariates were identified using a framework based on Directed Acyclic Graph (DAG) methodology, informed by the bioecological framework [44] (Fig. 1). This approach highlights the relationships between individual, microsystem, mesosystem, and macrosystem factors in understanding how experiences of violence at Wave 1 influence mental health outcomes at Wave 2.

All covariates were measured at Wave 1 and measured using standardised tools. Functional disability was measured by the Washington Group Short Set of 6 questions and was categorised as ‘yes’ if the participant responded as having any difficulty on at least 2 of the items and ‘no’ otherwise [45]. The number of reported meals the day before the survey was used as a proxy for food insecurity (less than one, two, three or more) as it reflects access to food and the adequacy of that access [46, 47]. Exposure to the intervention arm of the GSS at Wave 1 was recorded as a binary variable indicating receipt of any part of the intervention [48]. Data on age (continuous in years), romantic relationship status (yes/no) and sex was also included in the analysis.

### Statistical analysis

To address our first aim, we described depression and suicide attempts in late adolescence stratified by participant sociodemographics. To address our second aim to estimate the association between type of violence and (1) depression and (2) suicide attempts, we specified logistic regression models and estimated odds ratios. First, we estimated the crude odds ratio (Model 1, OR) followed by the adjusted odds ratio (Model 2, aOR), adjusting for the confounders discussed above. Robust standard errors were used to account for clustering by school. We conducted additional analyses to explore associations with lifetime exposure to each type of violence (physical, sexual, emotional). Sensitivity analyses were done using an alternative five categories (none, mild, moderate, moderately severe, severe) for the depression outcome utilising ordinal logistic regression [33, 49] (Table S2). We performed sex-stratified analyses for the association between lifetime exposure to each type of violence (physical, sexual, emotional) and mental health outcomes (depression, suicide attempts at Wave 2).

Our third aim was to examine if peer and family support modified the association between childhood violence and later in life mental health. We also conducted exploratory analyses to test for potential effect modification by peer and family connectedness on each subtype of violence and later in life mental health. We incorporated an interaction term into the logistic regression model with any violence, followed by each type of violence (emotional, physical, sexual). All analyses were conducted in Stata version 16.0.

## Results

### Sample characteristics

The sample for this study included 2773 participants, 1328 boys and 1445 girls. Table 1 describes the sociodemographic characteristics of the study sample. The median age was 13 years IQR (12, 14) at Wave 1 and 18 years IQR (16, 19) at Wave 2. 20.56% young people reported a disability at Wave 1. 45.2% (1252/2770) reported having three or more meals the previous day, 14.9% (415/2770) reported having had one meal the previous day, and 0.40% (11/2770) reported having no meal the previous day at Wave 1. 4.1% (113/2771) reported ever being in a relationship at Wave 1. 38.1% (1047/2751) reported low family connectedness at Wave 1, while 41.9% (1161/2769) reported low peer connectedness at Wave 1.

**Table 1:** Characteristics of 2773 adolescents by mental health outcomes in Luwero District, Uganda

Participant characteristics		Total			Depression (Wave 2)						Ever attempted suicide (Wave 2)					
					No			Yes (*n* = 342; 13.3%)			No			Yes (*n* = 119; 4.3%)		
		**n**	N	%	n	N	%	n	N	%	n	N	%	n	N	%
**Exposure**																
**Lifetime experience of violence (Wave 1)**																
Any violence		2,489	2,773	89.76	2,002	2,228	89.86	308	342	90.06	2,373	2,652	89.48	114	119	95.80
Any physical violence		2,409	2,773	86.87	1,936	2,228	86.89	294	342	85.96	2,300	2,652	86.73	107	119	89.92
Any physical violence excluding caning		1,898	2,773	68.45	1,509	2,228	85.69	252	342	73.68	1,803	2,652	67.99	93	119	78.15
Any sexual violence		174	2,773	6.27	125	2,228	5.61	40	342	11.70	155	2,652	5.84	19	119	15.97
Any emotional violence		1,576	2,773	56.83	1,239	2,228	55.61	218	342	63.74	1,492	2,652	56.26	82	119	68.91
**Effect modifiers**																
**Family connectedness (Wave 1)**																
Low		1,298	2,773	46.81	1,198	2228	53.77	174	342	50.88	1,419	2,652	53.51	56	119	47.06
High		1,475	2,773	53.19	1,030	2228	46.23	168	342	49.12	1,658	2,631	63.02	44	118	37.29
**Peer connectedness**																
Low		1,161	2,769	41.93	999	2228	44.84	158	342	46.20	1,179	2,652	44.46	70	119	58.82
High		1,608	2,769	58.07	1,229	2228	55.16	184	342	53.80	1,473	2,652	55.54	49	119	41.18
**Covariates**																
**Sex (Wave 1)**																
Male		1,328	2,773	47.89	1,115	2,228	50.04	127	342	37.13	1,282	2,652	48.34	44	119	36.97
Female		1,445	2,773	52.11	1,113	2,228	49.96	215	342	62.87	1,370	2,652	51.66	75	119	63.03
**Age**																
Wave 1 age (mean ± SD)		13 (± 1.53)		N/A	13 (± 1.55)		N/A	13 (± 1.54)		N/A	13 (± 1.54)		N/A	13 (± 1.55)		N/A
Wave 2 age (mean ± SD)		18 (± 1.76)		N/A	18 (± 1.77)		N/A	18 (± 1.75)		N/A	18 (± 1.77)		N/A	18 (± 1.75)		N/A
**Number of meals (Wave 1)**																
1 meal or less		426	2,770	15.38	336	2,226	15.09	70	341	20.53	399	2,649	15.06	26	119	21.85
2 meals		1,092	2,770	39.42	876	2,226	39.35	139	341	40.76	1,042	2,649	39.34	50	119	42.02
3 meals or more		1,252	2,770	45.20	1,014	2,226	45.55	132	341	38.71	1,208	2,649	45.60	43	119	36.13
**Exposure to Good Schools toolkit (Wave 1)**																
No		1,385	2,773	49.95	1,095	2,228	49.15	154	342	45.03	1,331	2,652	50.19	52	119	43.70
Yes		1,388	2,773	50.05	1,133	2,228	50.85	188	342	54.97	1,321	2,652	49.81	67	119	56.30
**Disability (Wave 1)**																
No		2,203	2,773	79.44	2,137	2,228	95.92	316	342	92.40	2,544	2,652	95.93	103	119	86.55
Yes		570	2,773	20.56	91	2,228	4.08	26	342	7.60	108	2,652	4.07	16	119	13.45
**Ever been in a relationship (Wave 1)**																
No		2,657	2,771	95.89	2,137	2,226	96.00	323	342	94.44	2,549	2,651	96.15	106	118	89.83
Yes		113	2,771	4.08	88	2,226	3.95	19	342	5.56	101	2,651	3.81	12	118	10.17

### Prevalence of violence in early adolescence

At Wave 1, the lifetime prevalence of any violence was 89.8% (2489/2773). There were no differences by sex with boys and girls experiencing similar levels of any lifetime violence (boys = 90.7% and girls = 88.9%). Lifetime physical violence was the most prevalent form of violence at 86.8% (2409/2773) overall; 87.8% (*n* = 1166) of boys and 86.0% (*n* = 1243) of girls. Most young people reported experiencing caning (total = 80.7%: 80.1% of girls and 81.5% of boys) and the prevalence of physical violence excluding caning was 68.4% (68.0% of girls and 69.0% of boys). The prevalence of lifetime emotional violence was 56.8% (1576/2771) and was 59.6% among boys (*n* = 791) and 54.3% among girls (*n* = 784). The prevalence of sexual violence was 6.3% (174/2771), which was higher among girls at 9.3% (*n* = 134) compared to 3.0% among boys (*n* = 40).

### Prevalence and socio-demographics of depression, suicide attempts

Overall, 13.3% (342/2570) of young people had PHQ-A scores indicative of depression at Wave 2 and 16.2% of girls (216/13344) had scores indicative of depression compared to 10.2% of boys (126/1236). 16.4% (88/537) of participants who had a functional disability had scores indicative of depression compared to 12.5% (254/2033) of those who did not have a disability. 4.3% (119/2771) of young people reported ever attempting suicide which was also higher among girls (5.2%) compared to boys (3.3%). Of the young people who had experienced any form of violence, 13.3% (308/2310) had PHQ-A scores indicative of depression and 4.5% (114/2487) reported ever attempting suicide. Of those who experienced sexual violence, 24.2% (40/165) had PHQ-A scores indicative of depression and 14.2% (19/134) reported ever attempting suicide (Table 1).

### Associations between violence in early adolescence and depression and suicide attempts in late adolescence

Overall, experiencing any violence in early adolescence (Wave 1) was not associated with later in life PHQ-A scores indicative of depression (Wave 2), including in sex-stratified models (Table 2). However, children who had experienced violence at Wave 1 had 2.6 times the odds of attempting suicide compared to those who had not experienced any form of violence (aOR: 2.50; 95% CI: 1.08, 6.27) and the odds of suicide attempts increased if caning was removed from the violence exposure (aOR: 3.02; 95% CI: 1.54, 5.92). In sex-stratified models, experiencing violence in early adolescence wasn’t associated with increased odds of attempting suicide among girls or boys (Table 2).

**Table 2:** Association between lifetime experience of any form of violence at Wave 1 and mental health outcomes (depression and suicide attempt) at Wave 2 for adolescents in Luwero district, Uganda, 2014–2018

	Crude			Adjusted*		
	OR	95% CI	n	aOR	95% CI	n
**Any violence including caning**						
**Depression (Wave 2)**						
Overall	1.02	(0.75, 1.40)	2,570	1.10	(0.78, 1.53)	2,564
Girls	1.25	(0.88, 1.79)	1,334	1.17	(0.81, 1.68)	1,331
Boys	0.79	(0.50, 1.26)	1,236	0.92	(0.53, 1.60)	1,233
**Suicide attempt (Wave 2)**						
Overall	2.68	(1.17, 6.15)	2,771	2.60	(1.08, 6.27)	2,765
Girls	3.11	(0.99, 9.72)	1,451	2.84	(0.87, 9.29)	1,448
Boys	2.20	(0.54, 8.90)	1,320	2.37	(0.58, 9.77)	1,317
**Any violence excluding caning**						
**Depression (Wave 2)**						
Overall	1.27	(0.96, 1.67)	2,570	1.30	(0.97, 1.74)	2,564
Girls	1.39	(1.00, 1.93)	1,334	1.31	(0.92, 1.85)	1,331
Boys	1.16	(0.74, 1.83)	1,236	1.26	(0.77, 2.06)	1,233
**Suicide attempt (Wave 2)**						
Overall	3.02	(1.54, 5.92)	2,771	2.83	(1.42, 5.65)	2,765
Girls	3.11	(1.00, 9.72)	1,451	2.84	(0.89, 9.29)	1,448
Boys	2.20	(0.54, 8.90)	1,320	2.37	(0.58, 9.77)	1,317

* Adjusted model includes: number of meals at Wave 1, receipt of GSS intervention at Wave 1, disability at Wave 1, age at Wave 1, sex at Wave 1 and romantic relationship status at Wave 1. n may not add up to 2771 due to missingness on outcome

### Exploratory analyses of the association between experience of different forms of violence in early adolescence and later in life mental health outcomes

We then explored associations for each type of violence. Experiencing any physical violence in early adolescence (Wave 1) was not associated with suicide attempts or depression in late adolescence. However, given the high prevalence of caning, once experiencing caning was removed from the construction of the physical violence variable, participants who had experienced physical violence excluding caning had higher odds of depression (aOR: 1.33; 95% CI: 1.04, 1.69) and suicide attempts (aOR: 1.67; 95% CI: 1.00, 2.79) compared to those that had not experienced physical violence (Table 3).

Young people who experienced emotional violence in early adolescence had higher odds of depression compared to those who had not experienced emotional violence (aOR: 1.46; 95% CI: 1.13, 1.88), and higher odds of having attempted suicide (aOR: 1.67; 95% CI: 1.00, 2.79). Finally, young people who had experienced sexual violence had higher odds of depression compared to those who had not experienced sexual violence (aOR: 1.81; 95% CI: 1.19, 2.74) and higher odds of having attempted suicide (aOR: 2.38; 95% CI: 1.39, 4.06) (Table 3).

**Table 3:** Association between lifetime experience of forms of violence at Wave 1 and mental health outcomes (depression and suicide attempt) at Wave 2 for adolescents in Luwero district, Uganda, 2014–2018

	Crude			Adjusted*		
	OR	95% CI	n	aOR	95% CI	n
**Physical violence (Wave 1)**						
Depression (Wave 2)	0.92	(0.66, 1.29)	2,570	0.97	(0.68, 1.39)	2,564
Suicide attempt (Wave 2)	1.36	(0.71, 2.61)	2,771	1.50	(0.77, 2.91)	2,765
**Physical violence excluding caning (Wave 1)**						
Depression (Wave 2)	1.33	(1.07, 1.66)	2,570	1.33	(1.04, 1.69)	2,564
Suicide attempt (Wave 2)	1.68	(1.01, 2.81)	2,771	1.67	(1.00, 2.79)	2,765
**Emotional violence (Wave 1)**						
Depression (Wave 2)	1.40	(1.11, 1.78)	2,570	1.46	(1.13, 1.88)	2,564
Suicide attempt (Wave 2)	1.72	(1.21, 2.45)	2,771	1.69	(1.16, 2.46)	2,765
**Sexual violence (Wave 1)**						
Depression (Wave 2)	2.23	(1.46, 3.41)	2,570	1.81	(1.19, 2.74)	2,564
Suicide attempt (Wave 2)	3.06	(1.81, 5.18)	2,771	2.38	(1.39, 4.06)	2,765

*Adjusted model includes: number of meals at Wave 1, receipt of GSS intervention at Wave 1; disability at Wave 1, age at Wave 1, sex at Wave 1, and romantic relationship status at Wave 1

### Sex-stratified analyses of the association between experience of different forms of violence in early adolescence and later in life mental health outcomes

In sex-stratified analyses, among boys, the experience of physical violence excluding caning, emotional or sexual violence was not associated with later in life suicide attempts or depression. However, girls who had experienced physical violence excluding caning had higher odds of depression compared to those that had not experienced physical violence excluding caning (aOR: 1.41; 95% CI: 1.07, 1.86) and higher odds of having attempted suicide (aOR: 2.60; 95% CI: 1.36, 4.94). Girls who had experienced emotional violence had higher odds of depression compared to girls who had not experienced emotional violence (aOR: 1.54; 95% CI: 1.15, 2.07) and higher odds of having attempted suicide (aOR: 1.73; 95% CI: 1.07, 2.79). Girls who had experienced sexual violence had higher odds of depression compared to girls who had not experienced sexual violence (aOR: 1.90; 95% CI: 1.25, 2.89) and higher odds of having attempted suicide (aOR: 2.45; 95% CI: 1.46, 4.31) (Table 4).

**Table 4:** Association between lifetime experience of any form of violence at Wave 1 and mental health outcomes (depression and suicide attempt) at Wave 2 stratified by sex for adolescents in Luwero district, Uganda, 2014–2018

	Crude			Adjusted*		
	OR	95% CI	n	aOR	95% CI	n
**Depression (Wave 2)**						
**Physical violence (Wave 1)**						
Girls	1.18	(0.82, 1.70)	1,334	1.11	(0.77, 1.59)	1,331
Boys	0.67	(0.42, 1.09)	1,236	0.74	(0.42, 1.31)	1,233
**Physical violence excluding caning (Wave 1)**						
Girls	1.49	(1.14, 1.95)	1,334	1.41	(1.07, 1.86)	1,331
Boys	1.16	(0.74, 1.81)	1,236	1.21	(0.74, 1.96)	1,233
**Emotional violence (Wave 1)**						
Girls	1.63	(1.21, 2.19)	1,334	1.54	(1.15, 2.07)	1,331
Boys	1.22	(0.83, 1.80)	1,236	1.29	(0.84, 1.98)	1,233
**Sexual violence (Wave 1)**						
Girls	2.13	(1.54, 3.47)	1,328	1.90	(1.25, 2.89)	1,325
Boys	0.97	(0.27, 3.54)	1,242	1.01	(0.27, 3.83)	1,239
**Suicide attempt (Wave 2)**						
**Physical violence (Wave 1)**						
Girls	1.60	(0.72, 3.57)	1,334	1.74	(0.70, 4.30)	1,331
Boys	1.09	(0.47, 2.52)	1,236	1.20	(0.55, 2.61)	1,233
**Physical violence excluding caning (Wave 1)**						
Girls	2.57	(1.37, 4.80)	1,334	2.60	(1.36, 4.94)	1,331
Boys	0.97	(0.48, 1.96)	1,236	0.90	(0.47, 1.75)	1,233
**Emotional violence (Wave 1)**						
Girls	1.85	(1.14, 2.30)	1,334	1.73	(1.07, 2.79)	1,331
Boys	1.64	(0.81, 3.32)	1,236	1.72	(0.83, 3.55)	1,233
**Sexual violence (Wave 1)**						
Girls	2.88	(1.65, 5.03)	1,445	2.45	(1.46, 4.13)	1,443
Boys	2.46	(0.76, 8.01)	1,326	2.82	(0.67, 7.82)	1,322

* Adjusted model includes: number of meals at Wave 1, receipt of GSS intervention at Wave 1, disability at Wave 1, age at Wave 1, and romantic relationship status at Wave 1

### Role of peer and family connectedness at Wave 1

In effect modification analyses, there was some evidence that peer connectedness modified the relationship between later in life depression and any violence, physical violence, and physical violence excluding caning. Among all adolescents who had low compared to high levels of peer connectedness at Wave 1, there were higher odds of PHQ-A scores indicative of depression for those who experienced the following forms of violence: any violence (aOR: 2.13; 95% CI: 1.04, 4.39, interaction term *p* = 0.032), physical violence (aOR: 1.70; 95% CI: 0.88, 3.28, *p* = 0.029), and physical violence excluding caning (aOR: 2.04; 95% CI: 1.30, 3.21, *p* = 0.033) at Wave 1. In contrast, among the adolescents who had high levels of peer connectedness at Wave 1, there were lower odds of PHQ-A scores indicative of depression for those who experienced: any violence (aOR: 0.69; 95% CI: 0.42, 2.13, *p* = 0.032), physical violence (aOR: 0.65; 95% CI: 0.41, 1.03, *p* = 0.029), and physical violence excluding caning (aOR: 0.97; 95% CI: 0.67, 1.41, *p* = 0.033) at Wave 1 (Table 5).

**Table 5:** Adjusted estimates of the odds ratio for the association between experience of violence in early adolescence and later in life depression, stratified by Wave 1 levels of peer connectedness

Violence type at Wave 1		High peer connectedness		Low peer connectedness										
	n	aOR	95% CI	aOR	95% CI	p-value for interaction	Wald statistic for interaction term	p-value for Wald statistic						
**Depression (Wave 2)**														
Any violence	2,564	0.69	(0.42, 2.13)	2.13	(1.04, 4.39)	0.032	4.61	0.032						
Physical violence	2,564	0.65	(0.41, 1.03)	1.70	(0.88, 3.28)	0.029	4.75	0.029						
Physical violence excluding caning	2,564	0.97	(0.67, 1.41)	2.04	(1.30, 3.21)	0.033	4.54	0.033						
Emotional violence	2,564	1.31	(0.93, 1.83)	1.58	(1.09, 2.29)	0.417	0.66	0.418						
Sexual violence	2,564	1.65	(1.06, 2.57)	1.70	(0.93, 3.10)	0.605	0.27	0.605						
**Suicide attempt (Wave 2)**														
Any violence	2,564	1.93	(0.55, 6.88)	3.36	(0.94, 12.10)	0.516	0.42	0.517						
Physical violence	2,564	1.00	(0.45, 2.23)	1.73	(0.65, 4.61)	0.324	0.97	0.324						
Physical violence excluding caning	2,564	1.09	(0.56, 2.11)	2.15	(1.09, 4.27)	0.111	2.53	0.111						
Emotional violence	2,564	1.55	(0.77, 3.15)	1.77	(1.07, 2.92)	0.724	0.12	0.724						
Sexual violence	2,564	3.65	(1.70, 7.87)	1.67	(0.86, 3.23)	0.323	0.98	0.323						

*Adjusted model includes number of meals at Wave 1, receipt of GSS intervention at Wave 1, disability at Wave 1, age at Wave 1, sex at Wave 1, and romantic relationship status at Wave 1

In sex-stratified analyses, among girls, peer connectedness did not modify the relationship between physical violence and depression or suicide attempts. However, among boys, peer connectedness modified the relationship between later-in-life depression and physical violence and physical violence excluding caning. Among the adolescent boys who had low levels of peer connectedness at Wave 1, there were higher odds of PHQ-A scores indicative of depression for those who experienced: physical violence (aOR: 1.85; 95% CI: 0.64, 5.42, *p* = 0.03), and physical violence excluding caning (aOR: 2.82; 95% CI: 1.27, 6.25, *p* = 0.01) at Wave 1. In contrast, among the adolescent boys who had high levels of peer connectedness at Wave 1, there were lower odds of PHQ-A scores indicative of depression for those who experienced: physical violence (aOR: 0.46; 95% CI: 0.23, 0.90, *p* = 0.03), and physical violence excluding caning (aOR: 0.77; 95% CI: 0.41, 1.43, *p* = 0.01) at Wave 1 (Table 6).

**Table 6:** Adjusted estimates of the odds ratio for the association between experience of violence in early adolescence and later in life depression, stratified by Wave 1 levels of peer connectedness and sex

	n	**High peer connectedness**		**Low peer connectedness**		**p-value for interaction**		
		aOR	95% CI	aOR	95% CI		Wald statistic for interaction term	p-value for Wald statistic
**Girls**								
Depression (Wave 2)								
Any violence (Wave 1)	1,332	0.77	(0.44, 1.35)	1.93	(0.83, 4.52)	0.141	2.16	0.141
Physical violence (Wave 1)	1,332	0.80	(0.46, 1.41)	1.60	(0.77, 3.33)	0.232	1.43	0.232
Physical violence excluding caning (Wave 1)	1,332	1.12	(0.72, 1.74)	1.77	(1.07, 2.92)	0.252	1.31	0.250
Emotional violence (Wave 1)	1,332	1.42	(0.95, 2.13)	1.69	(1.07, 2.66)	0.541	0.37	0.540
Sexual violence (Wave 1)	1,332	1.72	(1.10, 2.68)	1.96	(1.00, 3.85)	0.584	0.30	0.584
Suicide attempt (Wave 2)								
Any violence (Wave 1)	1,449	1.58	(0.33, 7.52)	4.72	(0.78, 28.77)	0.360	0.84	0.360
Physical violence (Wave 1)	1,449	0.98	(0.33, 2.86)	1.94	(0.67, 5.62)	0.293	1.11	0.293
Physical violence excluding caning (Wave 1)	1,449	1.76	(0.76, 4.07)	2.72	(1.19, 6.20)	0.364	0.83	0.364
Emotional violence (Wave 1)	1,449	1.93	(0.81, 4.58)	1.49	(0.75, 2.96)	0.739	0.11	0.739
Sexual violence (Wave 1)	1,449	3.50	(1.57, 7.81)	1.92	(0.95, 3.88)	0.387	0.75	0.387
**Boys**								
Depression (Wave 2)								
Any violence (Wave 1)	1,232	0.54	(0.25, 1.16)	2.75	(0.72, 10.46)	0.073	3.22	0.072
**Physical violence (Wave 1)**	**1**,**232**	**0.46**	**(0.23, 0.90)**	**1.85**	**(0.64, 5.42)**	**0.030**	**4.83**	**0.030**
**Physical violence excluding caning (Wave 1)**	**1**,**232**	**0.77**	**(0.41, 1.43)**	**2.82**	**(1.27, 6.25)**	**0.010**	**6.33**	**0.010**
Emotional violence (Wave 1)	1,232	1.15	(0.67, 1.10)	1.44	(0.80, 2.59)	0.546	0.36	0.546
Sexual violence (Wave 1)	1,232	1.00	(0.21, 4.53)	0.84	(0.17, 4.21)	0.937	0.01	0.937
Suicide attempt (Wave 2)								
Any violence (Wave 1)	1,316	2.63	(0.43, 15.97)	2.29	(0.35, 20.72)	0.913	0.01	0.913
Physical violence (Wave 1)	1,316	1.01	(0.45, 2.31)	1.54	(0.35, 6.73)	0.659	0.20	0.659
Physical violence excluding caning (Wave 1)	1,316	0.65	(0.29, 1.44)	1.61	(0.64, 4.01)	0.152	2.05	0.152
Emotional violence (Wave 1)	1,316	1.21	(0.46, 3.21)	2.71	(0.92, 8.04)	0.270	1.22	0.270
Sexual violence (Wave 1)	1,316	4.15	(1.04, 16.60)	1.00	(0.10, 9.98)	0.374	0.79	0.374

Family connectedness did not modify the association between any violence, physical violence or sexual violence in childhood and later in life depression or suicide attempts (Table 7), including in sex-stratified analysis (Table S3).

**Table 7:** Adjusted estimates of the odds ratio for the association between experience of violence in early adolescence and later in life depression and depression, stratified by Wave 1 levels of family connectedness

Wave 1 covariate		High family connectedness		Low family connectedness		*p*-value for interaction
	*n*	aOR	95% CI	aOR	95% CI	
**Depression (Wave 2)**						
Family connectedness						
Any violence	2,564	0.76	(0.44, 1.32)	1.45	(0.83, 2.55)	0.114
Physical violence	2,564	0.84	(0.52, 1.38)	1.03	(0.63, 1.68)	0.392
Physical violence excluding caning	2,564	1.28	(0.88, 1.84)	1.29	(0.90, 1.85)	0.842
Emotional violence	2,564	1.43	(1.00, 2.05)	1.42	(1.00, 2.05)	0.370
Sexual violence	2,564	1.27	(0.76, 2.13)	2.36	(1.30, 4.28)	0.427
**Suicide attempt (Wave 2)**						
Family connectedness						
Any violence	2,765	2.24	(0.87, 5.77)	2.89	(0.60, 13.91)	0.603
Physical violence	2,765	1.12	(0.46, 2.73)	1.52	(0.51, 4.55)	0.450
Physical violence excluding caning	2.765	1.19	(0.61, 2.31)	1.99	(1.01, 3.95)	0.194
Emotional violence	2,765	1.46	(0.75, 2.81)	1.91	(1.17, 3.11)	0.637
Sexual violence	2,765	2.59	(1.17, 5.75)	2.25	(1.05, 4.82)	0.947

*Adjusted model includes number of meals at Wave 1, receipt of GSS intervention at Wave 1, disability at Wave 1, age at Wave 1, sex at Wave 1, and relationship status at Wave 1

## Discussion

We drew on longitudinal data from 2771 young people growing up in Luwero, Uganda to address three study aims. First, we find a high prevalence of PHQ-A scores indicative of depression (13%) and of suicide attempts (4.3%) in late adolescence which were more prevalent among girls, among young people with disabilities and romantic relationship status at Wave 1. In this study’s setting, violence in early adolescence, when children were in primary school, was high with nearly 90% of children experiencing a form of physical violence, notably being hit with a cane at school, and nearly 70% of children experiencing emotional violence.

Our second aim was to test the relationship between violence in early adolescence and depressive symptoms and suicide attempts in late adolescence. We document a clear association between early life violence and lifetime suicide attempts reported in late adolescence, particularly among girls. All types of violence (physical, emotional, sexual) were associated with about a 2.5x increase in the odds of attempting suicide. We also find that any experience of violence in early adolescence was associated with higher odds of depression, but only once we removed caning from the measure of lifetime violence, which is a highly normative and widespread act of violence in Luwero, and in other parts of Uganda [50, 51]. In analyses stratified by types of violence, we found that physical violence excluding caning, emotional, and sexual violence had higher odds of later in life depression.

Our final aim examined the role of peer and family connectedness in the relationship between early life experiences of violence and later in life mental health. We found that there was a positive association between various types of violence and poor mental health at Wave 2 among those with low peer connectedness at Wave 1, but there was little evidence of an association among adolescents with high peer connectedness. Interestingly, and contrary to our hypothesis, family connectedness at Wave 1 did not modify the association between early violence and later poor mental health. However, where young people experienced low family connectedness, their early experiences of violence were associated with poorer mental health outcomes in later adolescence.

Most evidence on the effects of violence on mental health in LMICs is cross sectional; studies have focused on the impact of specific types of VAC, by perpetrator and setting, on the mental health of children. Findings show a positive association between experiences of violence and poor mental health outcomes among children [52–55]. A 2018 meta-analysis of 25 cross-sectional, 14 cohort, 6 case-control and 2 twin studies, reported that persons who experienced early childhood sexual violence had 1.89 times the odds of a suicide attempt compared to those who did not experience violence [56]. A 2019 meta-analysis of 106 studies observed similar odds ratios for the relationship between emotional violence and depression as well as for sexual violence and depression [57]. Studies in Uganda have also reported a similar association between childhood experience of physical, emotional, and sexual violence and attempting suicide [58, 59]. Our study adds to this body of literature by showing an association between an experience of any form of violence (physical, emotional, sexual) in early adolescence and lifetime suicide attempts and an association between several forms of violence in early adolescence and depression later in life.

Current scholarship offers several explanations for the links between violence and mental health, of which we highlight three. First, there are plausible neurobiological pathways by which early exposure to childhood violence can impact later life depression and suicide attempts through emotional and cognitive processes [60]. Experiencing traumatic events like violence in childhood is also linked to altered neuroendocrine stress responses that may play a role in the development of later life poor mental health [61]. Secondly, early experiences of violence are also associated with later life violence victimisation which can shape experiences of mental health. The prevalence of violence at Wave 2 was also high which may exacerbate the effects of early life violence on mental health and/or directly affect mental health, particularly PHQ-A scores which reflect symptoms in the past two weeks.

A third explanation comes from research on social norms which underscores how the normativeness of violence can affect later in life health outcomes as well as violence reporting. Social norms can be defined as “rules of action shared by people in a given society or group; they define what is considered normal and acceptable behaviour for the members of that group” [62]. It is possible that acts of violence that are common, and considered normative, like caning, are less likely to have a negative impact on the mental health of children that experience them. This is potentially because these acts may not be considered, processed, or reported as violence. This ‘cultural normativeness hypothesis’ is controversial, but authors have speculated that where acts of violence are normative, children exposed to these acts may be less likely to become as stressed and fearful in relation to these acts compared to non-normative acts of violence [63], thus lessening the likelihood of long-term adverse outcomes across the life course. Similarly, those performing the violent acts in contexts where they are normative may do so in a manner which may induce less fear and stress in children [63]. Conversely, the growing literature on the biological effects of prolonged exposure to stress underscore that bodies bear the physiological effects of exposure, regardless of whether we perceive these or not [64]. Our data suggest that including normative and common experiences of violence, such as caning, does attenuate the association between violence and later in life mental health outcomes. In Uganda, violence is reinforced by cultural norms which generally condone it [65]. For example, caning is a widespread and normative form of physical violence used to maintain or enforce positive behaviour among children [66, 67]. Further research is needed to confirm and further explore our findings and our results underscore the importance of considering how specific acts and types of violence shape the mental health trajectories of young people.

Our exploratory analyses indicated low peer connectedness may entrench the positive association between early life physical violence and depression, and that high peer connectedness may be protective against poorer mental health later in life. Although peer connectedness modified the association between any form of violence and physical violence, this was not the case for emotional and sexual violence. Children with high peer connectedness may be more likely to get support, validation for their feelings from friends shaping how they process experiences of violence and experience feelings of guilt, shame, and nervousness [68]. Sex-stratified analyses suggest that peer connectedness may play a protective role for boys experiencing physical violence in early adolescence, in relation to depression in later adolescence but not for girls. This differential effect in boys compared to girls could be due to gender differences in how peer support is internalized and how friendships function in adolescent development. Boys may rely more heavily on peer connections for social support outside the family, especially in situations involving physical violence. In contrast, girls may process physical violence differently or rely on a wider range of support systems, making peer connectedness less central as a modifying factor [69, 70]. Cross-sectional studies in Uganda and Brazil have found that support from peers and family was protective against mental health conditions, such as internalising problems and alcohol use disorder [28, 29]. Other studies have also reported that peer connectedness was protective from depression among children who had experienced violence in early adolescence and childhood [71–73]. Another study using CoVAC data found that school and peer connectedness may mitigate links between violence and other health outcomes, in this case, unintended pregnancy [43]. This finding highlights the need for interventions to strengthen peer relationships in early adolescence and the importance of measuring how young people’s peer and family networks shape their trajectories of mental health and wellbeing as young people grow up.

This study has both strengths and limitations. One limitation is that our sample is a school-based, recruited from 42 primary schools. However, the Ministry of Education, Uganda data suggests that about 14% of children of official primary school age in Uganda are out of school, and over half of those who enrol do not complete primary school [74]. This gap highlights the potential exclusion of vulnerable populations who may be at risk for violence and may differ significantly from the children in our study. As such, our findings are not generalisable beyond the sample included. Secondly, both violence and mental health outcomes are likely to be underreported and, it is likely that the true prevalence is higher than reported and the estimated ORs are biased towards the null. Although violence is measured in childhood and suicide attempts are measured four years later, both ask participants to recall lifetime exposures which limits our ability to ensure temporality. There may be residual confounding from unmeasured variables (e.g. attitudes about violence, social and gender norms, parenting). There are also limitations to the measures we use: the PHQ-A is not a clinical measure. It assesses depressive symptoms only over the past two weeks, emphasizing recent experiences rather than chronic mental health status. We also lack information on the timing or frequency of suicide attempts. The absence of baseline mental health measures limits our ability to control for pre-existing mental health issues at Wave 1. In addition, our measures of connectedness may not represent the multiple ways peers and parents affect children’s lives [75, 76]. There is a potential overlap between the perpetrators of violence and the protective factors being examined. In our sample, caregivers are one of the common perpetrators of childhood violence, which may directly influence a child’s sense of familial connectedness. Similarly, peers often play a role in both perpetrating and experiencing violence [75, 76]. This overlap may complicate the interpretation of our findings regarding protective factors, as a lack of connectedness may not only be a moderating factor but could also stem from negative experiences with those individuals who are supposed to provide support. Due to limitations in power, we were unable to conduct analyses based on the role of the perpetrator in cases of violence. Future research with larger sample sizes or targeted designs is recommended to explore how the identity of the perpetrator influences mental health. In addition, using the number of meals eaten the previous day as a proxy for food insecurity may not fully capture the complexity of food insecurity, as it reflects short-term food access rather than long-term patterns or other dimensions like meal quality or nutritional adequacy. Finally, the dichotomization of the connectedness variables could have resulted in a loss of power and within category information loss.

The main strength of this study is its longitudinal design, that allows for the estimation of associations between early life adverse exposures and later life mental health outcomes, particularly as this cohort is one of the few to examine both violence and mental health in a LMIC setting. We used validated measures for both exposure to violence and indications of depression.

Taken together, our findings suggest there are links between experiences of physical, emotional, and sexual violence in childhood and depression and suicide attempts later in life. These results both support and further the current global evidence base on links between violence and mental health outcomes. Further research should explore sub-types and groupings of violence, with a focus on severity, acceptability, and norms surrounding violence. Longitudinal studies measuring depression, anxiety, and suicidality alongside measures of violence should include measures for the severity, location, and perpetrators of violence to better identify opportunities for prevention and response.

A key implication of our findings is that early intervention to prevent violence can prevent mental health issues in adolescence and reduce suicidality. Our findings also suggest that, in addition to the health system, peer support systems may mitigate the effects of violence. Both violence and adverse mental health outcomes can be prevented. Integration of mental health and violence prevention, particularly in schools and homes, could interrupt or prevent the associations we document.

However, despite the adoption of the Child and Adolescent Mental Health Act in 2017 [77], in Uganda, funding for mental health services remains low [78, 79]. Furthermore, mental health services which are mostly provided through the primary health care system are inadequate. The country has a mere 53 psychiatrists, equating to approximately one psychiatrist per every one million people [80]. Outside of the primary health care system, there are few suicide prevention programs which are mostly run by non-government organisations [78]. According to existing literature, the majority of mental health services are delivered by traditional healers [81, 82]. Specifically, more than 60% of individuals seeking assistance from traditional healers are found to be experiencing moderate or severe mental health disorders [83].

A second implication relates to the need for youth friendly access to mental health services in peri-urban areas in Uganda. Efforts to integrate mental health and violence prevention, particularly in schools and homes, could interrupt or prevent the associations we document. Such efforts require engagement with funders and policy makers and engagement across sectors to fund and develop interventions and policies that seek to simultaneously prevent violence and improve the mental health of children and young people. Efforts to strengthen youth friendly health services that can respond to violence, depression, and suicide attempts are also central to preventing the adverse effects of violence and poor mental health as children grow up.

## Electronic supplementary material

Below is the link to the electronic supplementary material.

Supplementary Material 1

Supplementary Material 2

Supplementary Material 3

## Data Availability

The datasets upon which our findings are based belong to the London School of Hygiene and Tropical Medicine and Raising Voices, Uganda. For confidentiality reasons, the datasets are not publicly available. The datasets can however be availed upon reasonable request from the corresponding author with permission from the London School of Hygiene and Tropical Medicine and Raising Voices, Uganda.

## Abbreviations

aOR: Adjusted odds ratio
CADRI: Conflict in Adolescent Dating Relationships Inventory
GBD: Global Burden of Disease
CI: Confidence interval
CoVAC: Context of Violence Against Children
GSS: Good Schools Study
HIC: High income country
IHME: International Health Metrics and Evaluation
IQR: Interquartile range
LMIC: Low- and Middle- income countries
LSHTM: London School of Hygiene and Tropical Medicine
MRC: Medical Research Council
OR: Odds ratio
PHQ-A: Patient Health Questionnaire for Adolescents
UNICEF: United Nations Children’s Fund
UVRI: Uganda Virus Research Institute
VAC: Violence Against Children
