# Supplementary material for: Associations between violence in childhood, depression and suicide attempts in adolescence: evidence from a cohort study in Luwero district, Uganda
Source: BMC Public Health. 2024 Dec 18;24:3405. doi: 10.1186/s12889-024-20950-7 (PMC11653951; doi:10.1186/s12889-024-20950-7)
Supplement: Supplementary file 1 — Supplementary Material 1 [file 12889_2024_20950_MOESM1_ESM.docx]

Table S1. Measurement of violence variables at wave 1 (2014)

| variable | Type of violence | Items |
| --- | --- | --- |
| Care giver/ peer/ partner violence | Emotional | 1. insulted you, or called you rude or hurtful names 2. Accused you of witchcraft 3. Locked you out or made you stay outside? 4. Not given you food |
|  | Physical | 1. Twisted your arm or any other body part, slapped you, pushed you or thrown something at you 2. Punched you, kicked you, or hit you with a closed fist 3. Hit you with an object, such as a stick or a cane, or whipped you 4. Cut you with a sharp object or burnt you? |
|  | Sexual | 1. Disturbed or bothered you by making 2. sexual comments about you 3. Kissed you, when you did not want them to? 4. Touched your genitals or breasts when you did not want them to 5. Threatened or pressured you to do something sexual 6. made you have sex with them 7. had sex with you by physically forcing you |
| Teacher | Emotional | 1. Cursed, insulted, shouted at or humiliated you 2. Referred to your skin colour/ gender/ religion/ tribe or health problems you have in a hurtful way 3. Stopped you from being with other children to make you feel bad or lonely 4. Tried to embarrass you because you were an orphan or without a parent 5. Embarrassed you because you were unable to buy things 6. Stole or broke or ruined your belongings 7. Threatened you with bad marks that you didn’t deserve 8. Accused you of witchcraft |
|  | physical | 1. Hurt you or caused pain to you 3. Slapped you with a hand on your face or head as punishment 4. Slapped you with a hand on your arm or hand 5. Twisted your ear as punishment 6. Twisted your arm as punishment 7. Pulled your hair as punishment 8. Hit you by throwing an object at you 9. Hit you with a closed fist 10. Hit you with a stick 11. Caned you 12. Kicked you 13. Knocked you on the head as punishment 14. Made you dig, slash a field, or do other labour as punishment 15. Hit your fingers or hands with an object as punishment 16. Crushed your fingers or hands as punishment 17. Made you stand /kneel in a way that hurts to punish you 18. Made you stay outside for example in the heat or rain to punish you 19. Burnt you as punishment 20. Taken your food away from you as punishment 21. Forced you to do something that was dangerous 22. Choked you 23. Tied you up with a rope or belt at school 24. Tried to cut you purposefully with a sharp object 25. Severely beat you up |
|  |  | 1. Teased you or made sexual comments about your breasts, genitals, buttocks 2. Touched your body in a sexual way or in a way that made you uncomfortable 3. Showed you pictures, magazines, or movies of people or 4. children doing sexual things 5. Made you take your clothes off when it was not for a medical reason 6. Opened or took their own clothes off in front of you when they should not have done so 7. Kiss you when you didn’t want to be kissed 8. Make you touch their genitals, breasts or buttocks when you didn’t want to 9. Touch your genitals, breasts or buttocks when you didn’t want them to 10. Give you money/ things to do sexual things 11. Involve you in making sexual pictures or videos 12. Threaten or pressure you to have sex or do sexual things with them 13. Actually make you have sex with them by threatening or pressuring you, or by making you afraid of what they might do 14. Make you have sex with them by physically forcing you (have sex with you) |

For each variable Coded 1 if yes to any of the items and 0 if no to all the item
