# Supplementary material for: Associations between violence in childhood, depression and suicide attempts in adolescence: evidence from a cohort study in Luwero district, Uganda
Source: BMC Public Health. 2024 Dec 18;24:3405. doi: 10.1186/s12889-024-20950-7 (PMC11653951; doi:10.1186/s12889-024-20950-7)
Supplement: Supplementary file 2 — Supplementary Material 2 [file 12889_2024_20950_MOESM2_ESM.docx]

**Ologit models for depression**

Table S2. Association between lifetime experience of violence, and mental Health outcomes (depression) for adolescents in Luwero district, Uganda, 2014-2018.

|  | Crude | | | Adjusted* | | |
| --- | --- | --- | --- | --- | --- | --- |
|  | OR | 95% CI | n | aOR | 95% CI | n |
| Any violence | 1.27 | 0.98-1.65 | 2570 | 1.38 | 1.07-1.77 | 2564 |
| Physical violence | 1.14 | 0.93-1.40 | 2570 | 1.24 | 1.00-1.52 | 2564 |
| Emotional violence | 1.65 | 1.38-2.00 | 2570 | 1.75 | 1.46-2.09 | 2564 |
| Sexual violence | 2.08 | 1.37-3.17 | 2570 | 1.67 | 1.12-2.51 | 2564 |
