# Supplementary material for: Associations between violence in childhood, depression and suicide attempts in adolescence: evidence from a cohort study in Luwero district, Uganda
Source: BMC Public Health. 2024 Dec 18;24:3405. doi: 10.1186/s12889-024-20950-7 (PMC11653951; doi:10.1186/s12889-024-20950-7)
Supplement: Supplementary file 3 — Supplementary Material 3 [file 12889_2024_20950_MOESM3_ESM.docx]

| **Table S3: Adjusted estimates of the odds ratio for the association between experience of violence in early adolescence and later in life depression, stratified by wave 1 levels of family connectedness and sex** | | | | | | | | |
| --- | --- | --- | --- | --- | --- | --- | --- | --- |
| **Girls** | | | | | | | | |
|  | N | **High family connectedness** | | **Low family connectedness** | | **p, value for interaction** |  |  |
|  |  | aOR | 95%CI | aOR | 95%CI |  | Wald statistic for interaction term | p, value for Wald statistic |
| Depression (wave 2) | | | | | | |  |  |
| Any violence wave 1 | 1,322 | 1.3 | (0.63, 2.69) | 0.94 | (0.49, 1.83) | 0.497 | 0.46 | 0.5 |
| Physical violence wave 1 | 1,322 | 1.21 | (0.67, 2.18) | 0.96 | (0.51, 1.81) | 0.47 | 0.52 | 0.47 |
| Physical violence without cane wave 1 | 1,322 | 1.45 | (0.90, 2.34) | 1.29 | (0.84, 1.96) | 0.682 | 0.17 | 0.682 |
| Emotional violence wave 1 | 1,322 | 1.88 | (1.19, 2.99) | 1.28 | (0.76, 2.15) | 0.376 | 0.79 | 0.376 |
| Sexual violence wave 1 | 1,322 | 1.65 | (0.99, 2.75) | 1.88 | (0.98, 3.60) | 0.741 | 0.11 | 0.741 |
| Suicide attempt (wave 2) | | | | | | |  |  |
| Any violence wave 1 | 1,449 | 3.64 | (0.48, 27.67) | 2.06 | (0.44, 9.59) | 0.501 | 0.45 | 0.501 |
| Physical violence wave 1 | 1,449 | 2.39 | (0.48, 11.88) | 0.97 | (0.32, 2.95) | 0.221 | 1.5 | 0.221 |
| Physical violence without cane wave 1 | 1,449 | 2.94 | (1.07, 8.05) | 1.79 | (0.68, 4.72) | 0.397 | 0.72 | 0.397 |
| Emotional violence wave 1 | 1,449 | 1.71 | (0.83, 3.5) | 1.69 | (0.95, 3.00) | 0.793 | 0.07 | 0.793 |
| Sexual violence wave 1 | 1,449 | 1.37 | (0.54, 3.51) | 3.69 | (1.99, 6.84) | 0.12 | 2.41 | 0.12 |
| **Boys** | | | | | | |  |  |
| Depression (wave 2) | | | | | | |  |  |
| Any violence wave 1 | 1,232 | 1.3 | (0.63, 2.69) | 0.56 | (0.21, 1.50) | 0.187 | 1.74 | 0.19 |
| Physical violence wave 1 | 1,232 | 0.97 | (0.39, 2.41) | 0.46 | (0.19, 1.14) | 0.422 | 0.64 | 0.422 |
| Physical violence without cane wave 1 | 1,232 | 1.28 | (0.61, 2.71) | 1 | (0.54, 1.84) | 0.616 | 0.25 | 0.616 |
| Emotional violence wave 1 | 1,232 | 1.62 | (1.02, 2.55) | 0.91 | (0.47, 1.79) | 0.111 | 2.55 | 0.111 |
| Sexual violence wave 1 | 1,232 | 1.4 | (0.32, 6.20) | 0.66 | (0.09, 5.00) | 0.561 | 0.34 | 0.561 |
| Suicide attempt (wave 2) | | | | | | |  |  |
| Any violence wave 1 | 1,316 | 2.78 | (0.44, 17.62) | 1.9 | (0.25, 14.72) | 0.847 | 0.04 | 0.847 |
| Physical violence wave 1 | 1,316 | 1.08 | (0.44, 2.67) | 1.26 | (0.31, 5.16) | 0.882 | 0.02 | 0.882 |
| Physical violence without cane wave 1 | 1,316 | 0.83 | (0.39, 1.81) | 1.16 | (0.42, 3.20) | 0.545 | 0.37 | 0.545 |
| Emotional violence wave 1 | 1,316 | 1.69 | (0.63, 4.51) | 1.84 | (0.57, 5.91) | 0.791 | 0.07 | 0.791 |
| Sexual violence wave 1 | 1,316 | 5.52 | (1.14, 26.70) | 0.82 | (0.09, 7.50) | 0.242 | 1.37 | 0.242 |
